# On the value of meta‐research for early career researchers: A commentary

**DOI:** 10.1002/jcv2.12235

**Published:** 2024-04-24

**Authors:** Nicholas Fabiano, Arnav Gupta, Jess G. Fiedorowicz, Marco Solmi

**Affiliations:** ^1^ Department of Psychiatry University of Ottawa Ottawa Ontario Canada; ^2^ Department of Medicine University of Calgary Calgary Alberta Canada; ^3^ College of Public Health Kent State University Kent Ohio USA; ^4^ Department of Mental Health The Ottawa Hospital Ottawa Ontario Canada; ^5^ Ottawa Hospital Research Institute (OHRI) Clinical Epidemiology Program University of Ottawa Ottawa Ontario Canada; ^6^ Faculty of Medicine School of Epidemiology and Public Health University of Ottawa Ottawa Ontario Canada; ^7^ Department of Child and Adolescent Psychiatry Charité – Universitätsmedizin Berlin Berlin Germany

**Keywords:** early career researcher, meta‐research, open science, trainee

## Abstract

Meta‐research, also known as “research on research” is a field of study that investigates the methods, reporting, reproducibility, evaluation, and incentives along the research continuum. Meta‐research literacy is imperative to ensure high quality, transparent and reproducible primary data or meta‐research products. In this commentary, we propose that early career researchers should be trained in meta‐research as a foundation to develop a deeper understanding of the research process and ability to appraise the research literature and design high‐quality original studies, irrespective of their chosen field of study. We discuss the importance of meta‐research and open science from the perspective of an early career trainee, highlighting essential areas for growth and obstacles one may encounter.

**Key points**

**What's known?**

- Meta‐research, also known as “research on research” is a field of study that investigates the methods, reporting, reproducibility, evaluation, and incentives along the research continuum.

**What's new?**

- We discuss the importance of meta‐research and open science from the perspective of an early career trainee, highlighting essential areas for growth and obstacles one may encounter.

**What's relevant?**

- We propose that early career researchers should be trained in meta‐research as a foundation to develop a deeper understanding of the research process and ability to appraise the research literature and design high‐quality original studies, irrespective of their chosen field of study.

## BACKGROUND

In the ever‐evolving world of academia, it is essential to keep pace with new developments and trends. Each year, there are over 2 million scientific articles published, which makes navigating the intricacies of scholarly communication a daunting task even for experienced, large and well‐funded research groups (Ware & Mabe, 2012). This challenge poses an even greater impact on early career researchers, who are still exploring the norms and standards of their field while trying to establish their career path and build a strong research profile. To address these challenges and ensure that quality research is being communicated effectively to the academic community, the importance of meta‐research cannot be overstated. A “typical scientific experiment” follows the scientific method whereby one tests a hypothesis by carrying out an experiment and collecting data, which lead to specific conclusions, which either support or refute the original hypothesis. This differs from a “typical meta‐research project” whereby the investigator instead studies research itself, its production process and interpret it in an attempt to quantify how reliable its results and conclusions are. Meta‐research investigates the methods, reporting, reproducibility, evaluation, and incentives in the research process (Goodman & Dickersin, 2011). In evidence synthesis, meta‐research typically aims to confirm or confute findings from original studies, and to explore sources of heterogeneity and bias. This analysis is one of the pillars of evidence‐based medicine, since it allows for a complete evaluation of research practices, as well as research findings, for assessing credibility and certainty of results, resolving methodological discrepancies, improving reporting standards, ensuring reproducibility, and educating researchers on potential sources of bias across different study designs. Despite this, most researchers are not familiar with the field of meta‐research, and an even smaller fraction are formally trained in this area. Therefore, we propose that meta‐research should be incorporated into the curriculum for early career researchers, providing an opportunity for them to develop a deeper understanding of the research process and to gain insight into current trends, evidence gaps and best practices in their field. In this article, using an existing framework for meta‐research, we will discuss the various themes of meta‐research from the perspective of an early‐career trainee which are summarized in Table 1 (Ioannidis, 2018).

**TABLE 1:** Aspects of meta‐research that are relevant for trainees and early‐career researchers.

Methods: Designing and conducting research Comprehending differences in study designs, and determining the best study design for each research question. Learning different effect size measures, statistical significance, uncertainty measures, heterogeneity measures. Identifying “meta‐biases,” which are differences in results among collections of studies which cannot otherwise be appreciated at the individual study level. Understanding how to prepare a study protocol. Becoming familiar with library services, and studies' PDF retrieval. Gaining insight about data extraction and evidence synthesis techniques. Familiarizing with statistical softwares to conduct analyses. Gaining skills to become an effective team player and leader.
Reporting: Communicating research Understanding how research is disseminated in scientific publications or the media. Identifying and incorporating current bias‐preventing methods (e.g., protocol registration, adherence to reporting guidelines). Integrating results across studies and synthesizing available evidence. Quantifying compliance and effectiveness of transparency policies and data‐sharing methods.
Reproducibility: Verifying research Understanding the importance of replication of findings. Appraising methods and practice, and understanding how it influences reproducibility of results in future research. Quantifying false‐positive rates, small study effect, excess of significance bias. Quantifying publication bias.
Evaluation: Evaluating research Understanding the pyramids of evidence. Assessing credibility and certainty of observational and interventional evidence. Performing quality assessments of evidence. Identify sources of bias in the study design that might cause type I or type II errors. Identifying external factors (e.g., financial support, conflicts of interest, publication bias) that may affect results.
Researcher assessment/incentives: Hiring, promotion and tenure Understanding the importance of promoting high quality research, accounting for but going beyond merely the number of publications. Understanding the importance of publishing statistically “negative” findings. Being aware of incentives in the research system for career advancement. Understanding the value and limitations of traditional metrics such h‐index.
Open science: Making research accessible to all Becoming familiar with the concept of open science. The need to advocate for open science by changing the current scientific publishing model. Educating self with regards to UNESCO's recommendations about open science which have been accepted in all 193 supporting countries.

## METHODS

Early career trainees typically work in a specific project, collecting data for primary studies. They should ideally be able to put the individual study they are working on in the larger context of the available evidence and methodology, in order to go beyond the mere execution of assigned tasks, and the publication of results. It is imperative to become familiar with various clinical research study designs, ranging from primary data (e.g., randomized controlled trials [RCT], cohort studies, and case‐control studies) to evidence synthesis (e.g., systematic reviews, meta‐analyses, scoping reviews and umbrella reviews). This enables one to critically analyze study designs, to identify any sources of bias affecting primary data research projects trainees might be involved in, to avoid methodological mistakes, to be intentional in following transparent research principles, ultimately making the journey leading to the final results and publications a richer and more aware experience.

Emphasis must also be placed on understanding concepts such as effect size measures, uncertainty measures, statistical significance, and heterogeneity, which will then be applied to interpretation of findings of primary research projects. The effect size measures the strength of association between an exposure and outcome which determines the benefit or risk of a particular intervention, and can be used for continuous (standardized mean difference) or binary (relative risk, odds ratio [OR], or hazard ratio) data. To use the OR as an example: an OR > 1 indicates a greater odds of association between exposure and outcome; OR < 1 indicates a lower odds of association between exposure and outcome, compared with a control group. Thresholds exist to define an effect size very small, small, medium, large or very large. These effect sizes can be interpreted in the context of uncertainty measures. An uncertainty measure is a depiction of statistical dispersion of values which includes the range of possible values within which the true value lies and can include parameters such as 95% confidence interval (95% CI), 95% prediction interval (95% PI) and standard deviation. Statistical significance occurs when the conditional probability of the null hypothesis being true given the observed data falls under a predefined threshold, often 0.05 (Tenny & Abdelgawad, 2023). Different significance thresholds can be applied according to more stringent criteria on credibility of evidence (Solmi et al., 2023). Heterogeneity is often quantified by the *I* ^2^ or *τ* ^2^ statistic. *I* ^2^ can range from 0% to 100%, where larger values indicate more considerable heterogeneity present. In effect, this metric quantifies deviations in observed versus expected intervention effects that would be expected due to chance alone. The *p*‐value associated with this metric must also be taken into account since the measure of heterogeneity has low power when studies have small sample sizes or are few in number. Resultantly, although a statistically significant result indicates an issue with heterogeneity, a non‐significant result cannot entirely be taken as a lack of heterogeneity present, and is why a *p*‐value of 0.10 is occasionally used in these instances. *τ* ^2^ represents the between‐study variance of the true effect sizes, with *τ* ^2^ being the standard deviation of the true effect sizes (Cochrane Training). Notably, within meta‐research, one must acknowledge how effect sizes are an amalgamation of different studies and greater emphasis must be placed on the consistency of the direction of association, over magnitude itself as in primary studies. Finally, the clinical or public health relevance of each association should be interpreted in the context of the prevalence of the outcome of interest. For instance, on the relative scale, a small association between a risk factor and a highly prevalent condition will have higher public health implications than a large association with an extremely rare event. In order to become familiar with the methodology of meta‐research, early career trainees could consider using resources such as the Cochrane Training modules, the Cochrane Handbook for Systematic Reviews or the Meta‐Research Centre at Stanford, which provide an excellent overview of these concepts (Cochrane Training; METRICS).

In addition to these concepts, identifying “meta‐biases” is also a critical component of meta‐research that trainees must learn about. Meta‐biases are differences in results among collections of studies that cannot otherwise be appreciated at the individual study level (Goodman & Dickersin, 2011). These biases arise within secondary research such as systematic or umbrella reviews, and need to compare studies with different characteristics to measure their effect on the individual studies' findings (Henderson & Page, 2007). Moreover, various techniques exist to detect publication bias where studies with statistically significant positive results are published more compared to those with neutral or negative results (Sedgwick, 2015). Within syntheses of individual study findings, learners should familarize themselves with interpreting publication bias via funnel plots to understand the distribution and heterogeneity within published effect sizes, and small study effects where smaller studies tend to have larger effect sizes (Nuesch et al., 2010; Seagroatt & Stratton, 1998). Therefore, trainees must be aware of the potential for meta‐biases to impact available literature, and proactively take steps to strive to publish research findings, even in case of negative/non‐significant results.

In order to protect research integrity, an a priori study protocol highlighting the background, rationale, objectives and methodology of the project should always be prepared, and published or posted online before the research project starts. The creation of the protocol is of utmost importance since it holds researchers accountable to their initial objectives and methodology, and simultaneously informs the community of ongoing work to avoid duplication. The population, intervention, comparison, and outcomes framework should be used to structure a clearly defined research question (Schardt et al., 2007). The background section is where the early career researcher searches and summarizes the current available evidence on a topic. This is a crucial step since it allows for one to familiarize themselves with a specific scientific space and to identify any gaps present. Ideally, a systematic review of available literature should be conducted, to identify evidence gaps. It is important to consider the assistance of library services to ensure that searches used in a systematic review are broad enough to encompass all relevant data and to avoid bias. From here, the early career researcher can use the available evidence gathered, or lack thereof, to explain the rationale of conducting their current study while simultaneously citing the objectives which they aim to address. Next, the early career researcher must determine the appropriate methodology required to attempt to answer the objectives at hand. As previously described, here is where it becomes imperative to be familiar with the various study designs ranging from primary to evidence synthesis in order to determine which is most applicable for their specific scientific inquiry. Once a fitting study design is selected, the early career researcher can use this as a framework to highlight the specifics of the methodology such as data extraction, evidence synthesis and statistical analyses.

It is important to expose trainees to statistical analysis early in their careers, as it can be a daunting task if left until later stages. One must first have a basic understanding of statistical concepts acquired through didactic teaching in order to apply these concepts to real‐world research data. Then, trainees can explore more advanced statistical techniques through practical application by using software such as R or STATA, or more user friendly ones such as JAMOVI, SPSS, RevMan or CMA (R Core Team, 2021; RevMan; StataCorp, 2023; SPSS Inc, 2007; The jamovi project, 2023; CMA) to conduct their analyses in a “learn by doing approach,” which has been previously demonstrated to be effective (Weissgerber, 2021). This allows trainees to understand how to identify and correct analytical errors, as well as interpret statistical results in a critical way.

A significant portion of effectively carrying out a research project is determining how to simultaneously be both an effective team player and leader. In order to gain a complete understanding of the project, it is important that the leader not only oversees the methodology such as data extraction and statistical analysis, but also be an active team member in the process. This allows the leader to both assign tasks to other team members in order to maximize efficiency, but to also play an active role in the process, and lead by example. For example, when conducting a systematic review, the leader should actively partake in study screening, data extraction and statistical analysis. This provides the leader with real‐time perspective, better enabling them to address any obstacles or questions from more junior researchers which may arise. At the same time, this ensures the utmost quality of research output which is crucial for findings to be deemed valid. Further, the leader must emphasize organization above all. The steps of the research project must be clearly defined and all data extraction sheets must be easily interpretable to ensure the ubiquitous understanding amongst the group in order to minimize any potential errors which stem from lack of organization or misunderstanding. These skills may be innate for some; however, through practice and repetition, all early career researchers can incorporate these skills to become effective leaders.

## REPORTING

Knowledge of research reporting and effective communication is imperative to understand how science is disseminated in scientific publications or to the general public as a whole. As an early career trainee, it is crucial to develop a critical eye in evaluating research findings and not taking them at face value. Often, studies may be highly cited or appear in various media outlets, however, it is important to remember that this does not equate to overall study quality (Aksnes et al., 2019; Kunze et al., 2022). One must analyze the supporting evidence and strength of claims made by particular studies in an attempt to find any sources of bias which may influence the propagation of findings.

In particular, “reporting bias” occurs when statistically significant or positive results are preferentially disseminated (Page et al., 2014). In turn, this leads to the overestimation of treatment effects and potentially to misguidance of medical decisions, putting patients at risk (Driessen et al., 2015). Therefore preventative measures must be in place at each stage of the research continuum to prevent reporting bias. Before commencing a study, early career researchers should make it routine to prospectively register trials or publish protocols to promote transparency, prevent selective reporting, and increase the likelihood of publication (Chan et al., 2017).

Moreover, early career researchers should familiarize themselves with various reporting guidelines (e.g., Consolidated Standards of Reporting Trials [CONSORT], Strengthening the Reporting of Observational Studies in Epidemiology [STROBE], and Preferred Reporting Items for Systematic reviews and Meta‐Analyses [PRISMA]) which provide a framework for proper reporting and standardization of a specific study design of interest (Elm et al., 2007; Page et al., 2021; Schulz et al., 2010).

Recently, a large‐scale analysis of radiology conference abstracts and manuscripts found that neither accuracy estimates nor conclusion positivity were associated with acceptance rate (Frank et al., 2022). Therefore, at times, reporting bias may occur due to factors prior to submission such as nonsubmission due to unfavorable results rather than at the editorial level (Song et al., 2014). Therefore, we encourage early career researchers to submit their studies for publication on the basis of strong research practices, and to not be discouraged by non‐significant or non‐positive findings.

## REPRODUCIBILITY

Early career trainees should additionally understand factors influencing the reproducibility of studies' results. Theoretically, given a single set of data and methods, a second researcher should be able to reproduce similar results as the original author. However, this may not be the case as there have been numerous instances where effect sizes in meta‐analyses have significantly varied upon recalculation, for instance (Lakens et al., 2016), or when significant findings have not been replicated in additional observational studies.

Reproducible research is important as it allows individuals to build upon existing knowledge, analyze new subsets of patient populations or data, and appraise the robustness of prevailing conclusions (Lakens et al., 2016). Given the importance of reproducibility in cumulative science and the requirement of consistency in causation research, it is important to integrate training on reproducibility in early career research curricula.

Problems in reproducibility frequently stem from the extent to which authors are transparent about their study's data, methods, or protocol (Ioannidis, 2018). As error rates have been shown to be high among even experienced researchers, early career trainees should be extra prudent when synthesizing individual study data. Augmented by the lack of openly‐available meta‐analytic data sets or programming code used in a given publication, we encourage early career researchers to do so with their own research projects, while being unafraid to reach out to primary source authors to make clarifications about data collection or synthesis methods (Lakens et al., 2016). On the other hand, large volumes of coding and statistics are quite prone to error, especially when performed by a single individual, leading to non‐reproducible results (Lakens et al., 2016). While cumbersome, we encourage early career researchers to perform analyses in duplicate to both verify their results and consolidate knowledge of data synthesis methods for future projects through peer support. Using different software can also be considered. As beginners, early career researchers will not always be aware of best practices; thus, when building protocols, it is important to recruit experts (e.g., librarians) to assist with different steps of their projects, complete high quality protocols, and ultimately ensure reproducibility. Finally, the phrase “data is available upon request” has become commonplace; however, the majority of researchers do not follow through with these claims (Chawla, 2023). Instead, incentives should be in place to prioritize making data publicly available to aid in reproducibility and minimize the waste of valuable resources. This also allows for early career researchers to analyze data themselves, interpret the results, and strengthen their understanding of research methods and statistical analyses. In some instances, this has even led to students finding errors in published studies from prominent researchers, showing the importance of transparency and data availability (Page et al., 2021).

Currently, there is a lack of reproducibility‐related meta‐research (Hardwicke et al., 2020). Recently, López‐Nicolás et al.'s (2022) systematic review of meta‐reviews in clinical psychology sampled 100 meta‐analyses from 2001 to 2020 to identify predictors of reproducibility‐related indicators and most frequently spoke about lack of transparency or data‐sharing. We recommend both early and advanced researchers contribute to meta‐research in reproducibility through overviews or umbrella reviews of replication studies to adapt recommendations to field‐specific factors. Additionally, we recommend that meta‐research continues to find objective ways of determining compliance with reporting standards, quantifying standards, and mitigating false‐positives to assist early career researchers. With its proliferation, we expect better compliance and dissemination of higher quality, consistent results.

## EVALUATION

Evaluation of research primarily involves assessing the extent of bias, precision, and applicability to clinical practice (Verhagen et al., 2001). While quantitative synthesis provide a unified effect size, somewhat accounting for publication bias, qualitative syntheses remain valuable as research evaluation contextualize their results (Carroll & Booth, 2015). This process requires considerable skill to not simply list the results of various studies but to integrate and synthesize the available literature to the reader in a meaningful way. In fact, Hannes and Macaitis (2012) found that the majority of qualitative syntheses among a 20‐year sample included some evaluative component. Especially in secondary research (e.g., systematic reviews, umbrella reviews), evaluation is a vital component in translating statistical significance to clinical significance. Early career researchers should have a strong understanding of various tools to bolster their own research.

However, some challenges exist for research evaluation. Despite being advantageous, the sheer volume of research evaluation tools may be overwhelming. Even within a single paper, individuals may use different tools with different criteria which can affect how results are appraised. Rather than attempting to learn all the tools, we recommend early career researchers familiarize themselves with the pyramid of evidence (i.e., across different study types), the basics about biases (e.g., selection bias, reporting bias, publication bias), compare and contrast their knowledge with the existing tools, and develop an approach to critically appraise papers. We especially recommend that early career researchers take initiative in quality assessment portions of secondary research to directly apply this knowledge and identify sources of bias contributing to type I error (false‐positive) and type II error (false‐negative). Previous meta‐research has described how arbitrary cut‐offs increase the impact of type I and type II error as tools like *p*‐values (e.g., use these to quantify magnitude of effect size) and confidence intervals (e.g., interpreting whether or not the null hypothesis falls within the range) are misinterpreted. Rather, understanding the definition of such concepts, understanding what information they provide is more valuable. Early career researchers can thus appropriately interpret results, understand which variables within their equations are prone to bias, understand what steps can be taken to produce bias‐free, accurate estimates (Rothman, 2010). Additionally, in conjunction with the recommendation of establishing a priori protocols, should early career researchers wish to perform sensitivity analyses in secondary research, we encourage them to remain objective by having preset criteria for exclusion. While it may be difficult to anticipate every possible scenario during data synthesis, this both minimizes reporting bias, and ensures research is evaluated carefully and meaningfully (Carroll & Booth, 2015).

There are currently various frameworks for research evaluation. The most notable example are The Grading of Recommendations Assessments, Development, and Evaluation (GRADE) guidelines and the Ioannidis criteria for certainty or credibility of evidence (Guyatt et al., 2011). Also, the quality of meta‐analyses can be assessed with A Measurement Tool to Assess Systematic Reviews tool (Shea et al., 2017). By accounting for evidence type and various factors (e.g., risk of bias, publication bias), among other criteria, both are important for translating evidence from systematic reviews to clinical guidelines (Guyatt et al., 2011). Likewise, for primary research, there are tools for different levels of evidence ranging from RCTs (e.g., Jadad scale, Cochrane Risk of Bias tools) to case series (e.g., JBI tools) (Halpern & Douglas, 2005; Munn et al., 2019). Meta‐research should focus on the extent to which these guidelines are adhered, and in what ways, early career researchers may both familiarize themselves with and implement them in their own endeavors, such as designing their own primary studies.

Another field within meta‐research we recommend exploring is examining how financial and non‐financial sources or conflicts of interest influence biasedness of results. While reporting guidelines like PRISMA mandate reporting biases, there have been reports where effect sizes have varied depending on the first author's conflicts (Lakens et al., 2016). Continued meta‐research in such contentious topics may better inform early career researchers who may not have the experience to contextualize such nuances. As meta‐research continues to quantify bias and heterogeneity due to various study‐specific or methodological factors, early career researchers can develop more comprehensive, objective critical appraisal skills to assess the quality of evidence while understanding how to initiate high quality projects.

## INCENTIVES

One important aspect of research is rewarding early researchers to encourage adherence to best practice standards and reward a passion for science (Ioannidis, 2018). Irrespective of workplace, long‐term incentive programs have been associated with a 1.28‐fold increase in performance (Condly et al., 2008). Although early career researchers may not frequently be involved in leadership positions, a clear understanding of incentives should be in place that favors and rewards meta‐research productivity and initiatives.

There exists a wide range of meta‐research in incentives for both consequences and rewards. For example, Li et al. (2021) identified common forms of research misconduct within RCTs, and provided a checklist and suggestions for authors and reviewers to mitigate misconduct. Likewise, Resnik (2015) explored how discordance in wording for national research policies may be a source of research misconduct and lack of accountability. Meanwhile, there is a proliferation of research for quantifying productivity. For instance, while quantifying productivity introduced some “objective” parameter rewarding scientific productivity, bibliometric indexes complement but can't replace a qualitative evaluation of research. For instance, the H‐index, the number where a researcher's *h* papers have been cited *h* times, is considered to be widely misunderstood and meta‐research has criticized its poor predictive validity due to poor assumptions regarding impact (Barnes, 2017). Rather, authors like Hicks (2012) have highlighted how there needs to be more research examining performance‐based university funding systems to quantify and reward quality research (e.g., robust methods) (Bornmann & Daniel, 2009; Hicks, 2012).

This is in keeping with more meta‐research encouraging the dissemination of negative results. As an early career researcher, negative results may be disappointing as previous meta‐research has demonstrated that positive clinical trial results have a greater chance of publication then negative clinical trial results, which may limit the ability to build strong research profiles (Johnson & Dickersin, 2007). Rather, in his commentary, Taragin (2019) emphasized how negative findings encourage transparency within research, better explain variation between studies evaluating similar outcomes, create more robust models, and even regain the public's trust in this era of misinformation. While daunting, the ability to encourage discourse with conflicting results will allow early researchers to find more opportunities to meaningfully contribute to growing bodies of literature, hone their research skill sets, and develop positive habits towards robustly performed studies.

Altogether, exploratory meta‐research is a valuable first step, but more validation research for solutions is necessary to help supervisors identify the most optimal way of rewarding research and discouraging misconduct. Early career researchers may also benefit from such research as they can subsequently identify metrics to objectively measure their research proficiency, and take directed steps for self‐growth.

## OPEN SCIENCE

Early career researchers should familiarize themselves with the concept of open science, which aims to make research transparent and accessible to everyone for the benefits of society and science. Open science occurs at all stages of the research continuum, from creation of the research question to publication and dissemination of findings (Government of Canada I, 2022). When creating the research question, early career researchers should create openly accessible protocols for their study in order to maximize community engagement. This allows the early career researcher to get real‐time feedback from experts in their respective field to strengthen their own knowledge on a topic and ensure that their methodology is sound. From here, data collection and analysis must encompass methods which are clear and reproducible. Once the data for a given study is collected, it is crucial that data is openly available to allow for public scrutiny and avoidance of research waste. During the publication process, emphasis must be placed on making manuscripts as easily accessible as possible. This can occur even prior to publication, whereby manuscripts are uploaded to pre‐print servers, which allow for unrestricted access to non‐peer reviewed articles. When selecting a journal for publication, early career researchers should emphasize those which offer open access models in order to avoid their findings being lost behind a paywall which is not accessible to all. This ensures that the knowledge acquired from their research can be optimally shared with the scientific community in order to advance the field and fuel future research questions. For a more complete overview of the value and implications of open science, early career researchers should refer to the recommendations created by the United Nations Educational, Scientific and Cultural Organization, which have been accepted in all 193 supporting countries (UNESCO).

## CONCLUSION

Training early career researchers in meta‐research has the potential to boost their methodological insight on research, preparing them to conduct sound research collecting primary data after having identified evidence gaps, developing critical interpretation skills of available evidence, and at the same time jump‐starting their high quality scientific productivity and creating future independent scientists and leaders.

## AUTHOR CONTRIBUTIONS

**Nicholas Fabiano**: Conceptualization; project administration; supervision; writing – original draft; writing – review & editing. **Arnav Gupta**: Conceptualization; project administration; supervision; writing – original draft; writing – review & editing. **Jess G. Fiedorowicz**: Supervision; writing – original draft; writing – review & editing. **Marco Solmi**: Supervision; writing – original draft; writing – review & editing.

## CONFLICT OF INTEREST STATEMENT

MS received honoraria/has been a consultant for Abbvie, Angelini, Lundbeck, Otsuka, unrelated to this work.

## ETHICAL CONSIDERATIONS

None.

## Data Availability

Data sharing is not applicable to this article as no new data were created or analyzed in this study.
